# Epidemiology, Virulence Factors, and Antimicrobial Resistance in *Staphylococcus aureus*

**DOI:** 10.3390/antibiotics14080792

**Published:** 2025-08-04

**Authors:** Maria de Lourdes Ribeiro de Souza da Cunha

**Affiliations:** Department of Genetics, Microbiology and Immunology, Institute of Biosciences, São Paulo State University (UNESP), Botucatu 18618-691, SP, Brazil; mlrs.cunha@unesp.br

Serious infections caused by bacteria that are resistant to commonly used antibiotics have become a global health problem in the 21st century. An important example is the methicillin-resistant *Staphylococcus aureus* (MRSA), which poses a serious threat to public health worldwide because of the rapid spread and diversification of pandemic clones, which are characterized by increasing virulence and antimicrobial resistance [1].

Antibiotic resistance, which was initially a problem faced by hospital settings associated with an increased number of hospital-acquired infections, usually in critically ill and immunocompromised patients, has now spread to the wider community, causing serious illness in previously healthy and conventionally non-vulnerable patients [2]. In the case of community-acquired infections, population characteristics, living conditions, agglomerations, underlying diseases, injectable drug use, the presence of insulin-dependent diabetes, and the frequency of antibiotic use contribute to the selection of resistant bacteria. Furthermore, we continue to fail to contain the spread of resistance genes, compromising community healthcare. Community-associated (CA)-MRSA usually occurs in individuals who have not been hospitalized or undergone medical procedures such as dialysis, surgery, or catheterization in the year prior to infection, all of which are common factors in hospital-acquired MRSA (HA-MRSA) infections [3].

Infections caused by *S. aureus* are usually severe because of the production of various virulence factors, including extracellular enzymes, cytolytic toxins, and superantigen toxins. The arsenal of virulence factors of *S. aureus* is extensive; some of them are part of the cell structure itself and others are extracellular factors that are produced during growth and excreted to the extracellular medium. Both types of virulence factors play important roles in the pathogenesis of infection [4].

The typical cultural behaviors of different populations, such as overcrowded living conditions, compromised healthcare, and poor hygiene, may be more relevant in the pathogenesis of some forms of *S. aureus* infections. Within this context, natives undeniably belong to the risk group for carrying resistant microorganisms and are susceptible to both the acquisition and spread of infections [5].

The study by Abraão et al. (2023) investigated the prevalence and risk factors for nasal and oral carriage of methicillin-sensitive *S. aureus* (MSSA) and MRSA among indigenous communities in northern and southeastern Brazil. The authors evaluated genetic diversity, dissemination, virulence factors, and antimicrobial resistance associated with ethnic, demographic, environmental, and behavioral factors. A total of 400 Indians (from near-urban areas and remote hamlets) were screened for *S. aureus* and CA-MRSA colonization. The study revealed a higher prevalence of *S. aureus* carriage among Shanenawa ethnicity individuals (41.1%). Thus, ethnicity appears to be associated with the prevalence of *S. aureus* in these populations. CA-MRSA was found in three isolates (0.7%), all of them SCC*mec* type IV. PFGE analysis identified 21 clusters among the *S. aureus* isolates and MLST analysis showed a predominance of sequence type 5 among these isolates.

Considering a completely different perspective, people living with HIV/AIDS must be cited as a risk group. Studies conducted in different countries demonstrated significant colonization of this population with MRSA, a finding that can be attributed in part to their intense contact with health services [6]. In the study by Obanda et al. (2022) investigating abattoir workers, MRSA carriage was higher in HIV-positive individuals (24/89, 27.0%) than in HIV-negative participants (94/648, 14.5%; *p* = 0.003). The prevalence of MRSA carriage (0.4%) identified in that study was low compared to studies conducted with abattoir workers in Europe (5.6%) [7] and the United States (3.6%) [8]. However, the low rate is consistent with another study investigating MRSA carriage in Kenya (0.8%) [9].

Studies found that individuals carrying a high bacterial load have a six times greater risk of developing staphylococcal infection than non-carriers or individuals with a low bacterial load [10]. This phenomenon seems to be even more common among MRSA carriers [11]. Although the nasopharynx is the most consistent site of colonization by *S. aureus* and has been indicated as the most appropriate site for swab sampling, other sites (extra-nasal) can also be colonized. Recent studies demonstrated that a substantial number of individuals, ranging from 7% to 32%, are exclusive *S. aureus* carriers in the oropharynx, suggesting that the inclusion of a throat swab in addition to a nasal swab may be important for the success of surveillance programs [12].

The study by Silva et al. (2022) investigated the prevalence and factors associated with the nasal, oral, and rectal carriage of *S. aureus* and MRSA in bedridden patients and residents of long-term care facilities for the elderly (LTCFs) in Botucatu, SP, Brazil. The prevalence of *S. aureus* and MRSA was 33.6% (*n* = 76) and 8% (*n* = 18), respectively. At the nine LTCFs studied, the prevalence of *S. aureus* ranged from 16.6% to 85.7% and that of MRSA from 13.3% to 25%. The study showed a high prevalence of *S. aureus* among elderly residents of small (<15 residents) and medium-size (15–49 residents) LTCFs, as well as a higher prevalence of MRSA in the oropharynx.

The contributions to this Special Issue on clinical MRSA isolates include the following studies: Boutet-Dubois et al. (2023) described the phenotypic and genotypic evolution of MRSA strains that became resistant to daptomycin (DAP) in two unrelated patients with bacteremia treated with this antibiotic in two hospitals in the South of France. DAP MICs were determined using the broth microdilution method on the pairs of isogenic (DAP-S/DAP-R) *S. aureus* isolated from bloodstream cultures. Whole-genome sequencing was carried out using the Illumina MiSeq Sequencing system. The two cases revealed DAP-R acquisition by MRSA strains within three weeks in patients treated with DAP. The study highlights the non-systematic cross-resistance between DAP and glycopeptides. The use of DAP as first-line therapy at optimal dosages must be considered when patients are at risk of MRSA infection. Moreover, it is crucial to monitor DAP MIC in persistent MRSA bacteremia. Kmiha et al. (2023) studied the genetic lineages, antibiotic resistance genes, and virulence determinants of *S. aureus* isolates from clinical samples of burn patients in Tunisia. All isolates from the clinical samples of burn patients were confirmed as MRSA, with high rates of resistance to ciprofloxacin and gentamicin conferred by different antibiotic resistance genes. The data revealed the presence of resistance genes and a different virulence profile in MRSA isolates. Tălăpan et al. (2023) evaluated the frequency of isolation of *S. aureus* from different pathological samples in Romania in order to establish the ratio of MRSA to MSSA strains and the antibiotic resistance profile of the isolated microorganisms. Up to 39.11% of *S. aureus* strains were resistant to oxacillin (MRSA), with 49.97% being resistant to erythromycin and 36.06% showing inducible resistance to clindamycin. Resistance rates to ciprofloxacin, rifampicin, gentamicin, and trimethoprim-sulfamethoxazole were 9.98%, 5.38%, 5.95%, and 0.96%, respectively. There was no resistance to vancomycin. Between 2017 and 2022, the percentage of MRSA strains decreased from 41.71% to 33.63% but sharply increased to 42.42% in 2021 (the year of the COVID-19 pandemic, when the percentage of strains isolated from lower respiratory tract infections was higher than that of strains isolated from wounds or blood, as in previous years). Quezada-Aguiluz et al. (2022) aimed to detect and characterize resistance to macrolides, lincosamides, and type B streptogramins among HA-MRSA and CA-MRSA isolates collected between 2007 and 2017 within the *S. aureus* surveillance program of the National Institute of Public Health of Chile (ISP). Most of the HA-MRSA isolates (97%) were resistant to clindamycin, erythromycin, azithromycin, and clarithromycin. Among CA-MRSA isolates, 28% were resistant to erythromycin and azithromycin and 25% to clarithromycin. The *ermA* gene was the predominant gene identified among these isolates.

*Staphylococcus pseudintermedius* is an opportunistic pathogen frequently isolated from canines [13]. Suepaul et al. (2023) focused on isolates obtained from healthy dogs and their owners who presented at clinics for routine veterinary care and used whole-genome sequencing-based analyses for strain comparisons. A total of 25 humans and 27 dogs were sampled at multiple sites, yielding 47 and 45 isolates, respectively. Whole-genome sequence analysis was performed. The virulence content did not provide insights toward a tendency of colonization of humans but supported that there may be differences in the surface proteins between carrier strains and those causing pyoderma. The study identified 13 cases in which humans were infected with strains from the dog they owned.

The cited studies are relevant since they suggest that *S. aureus* carriers are at higher risk of acquiring infection and are an important source of dissemination of bacteria among individuals. A comprehensive approach involving the special populations described above that combines epidemiological strategies with genetic characterization of staphylococci may provide insights into the genesis and dissemination of MRSA strains.
